# Malnutrition: The Importance of Identification, Documentation, and Coding in the Acute Care Setting

**DOI:** 10.1155/2016/9026098

**Published:** 2016-09-28

**Authors:** Jane Kellett, Greg Kyle, Catherine Itsiopoulos, Mark Naunton, Narelle Luff

**Affiliations:** ^1^Faculty of Health, University of Canberra, Canberra, ACT 2601, Australia; ^2^School of Clinical Sciences, Queensland University of Technology, Brisbane, QLD 4000, Australia; ^3^School of Allied Health, LaTrobe University, Bundoora, Melbourne, VIC 3086, Australia; ^4^ACT Health, Garran, Canberra, ACT 2605, Australia

## Abstract

Malnutrition is a significant issue in the hospital setting. This cross-sectional, observational study determined the prevalence of malnutrition amongst 189 adult inpatients in a teaching hospital using the Patient-Generated Subjective Global Assessment tool and compared data to control groups for coding of malnutrition to determine the estimated unclaimed financial reimbursement associated with this comorbidity. Fifty-three percent of inpatients were classified as malnourished. Significant associations were found between malnutrition and increasing age, decreasing body mass index, and increased length of stay. Ninety-eight percent of malnourished patients were coded as malnourished in medical records. The results of the medical history audit of patients in control groups showed that between 0.9 and 5.4% of patients were coded as malnourished which is remarkably lower than the 52% of patients who were coded as malnourished from the point prevalence study data. This is most likely to be primarily due to lack of identification. The estimated unclaimed annual financial reimbursement due to undiagnosed or undocumented malnutrition based on the point prevalence study was AU$8,536,200. The study found that half the patients were malnourished, with older adults being particularly vulnerable. It is imperative that malnutrition is diagnosed and accurately documented and coded, so appropriate coding, funding reimbursement, and treatment can occur.

## 1. Introduction

Malnutrition has been identified as a significant clinical problem in hospital settings both nationally and internationally [1, 2]. Malnutrition adversely affects physical well-being, interferes with health treatments, and increases healthcare costs [2]. The reported prevalence of malnutrition in recent Australian studies is 12–53% in acute settings [3–8].

Malnutrition has been defined by the American Society for Parenteral and Enteral Nutrition as cited in Mueller et al. [9] as “an acute, subacute or chronic state of nutrition, in which varying degrees of overnutrition or undernutrition with or without inflammatory activity have led to a change in body composition and diminished function.” For the purpose of this paper, the term malnutrition will refer to undernutrition.

Malnutrition directly impacts health care costs and is captured in the Activity-Based Funding model used for the funding of hospitals in Australia [10]. Activity-Based Funding is an output based funding model which funds a health care service for the cost of patient care. The amount of funding is dependent on the accurate documentation of all relevant diagnoses and the health care activities associated with patient care during an admission in the patient clinical records and discharge summary [10]. When a patient is discharged, their medical notes are audited by medical coders. A Diagnosis Related Group (DRG) is assigned based on the patient's major diagnosis, surgeries, comorbidities, complications, and other interventions recorded [11]. Hospitals are subsequently reimbursed for the patient admission based on this DRG. Malnutrition, when documented as a comorbidity or complication, has the ability to influence a DRG, often resulting in a higher classification which has the potential to attract greater hospital reimbursement [12].

Several previous Australian studies have reported estimations of unclaimed reimbursements for patient admissions, where malnutrition was not recorded as a comorbidity as part of the DRG [6, 7, 13–15]. In 2009, Melbourne study estimated an annual deficit to the hospital in reimbursements of $1,850,540 for undiagnosed or undocumented cases of malnutrition [14]. A similar study conducted in Brisbane estimated annual unclaimed expenses of $1,677,235 due to undiagnosed and undocumented malnutrition [15].

The Australian Capital Territory (ACT) is a territory within Australia, where Canberra the capital city of Australia is situated. The estimated population of the ACT is currently 390,800 [16]. However, the tertiary teaching hospital in this region supports a population of almost 540,000 as it is a referral centre providing a range of specialist services to both ACT region and people living outside of the ACT in the South Eastern Region of New South Wales (NSW) [17].

As there are currently no data on malnutrition in adults residing in the ACT, the aim of this study was to determine the prevalence of malnutrition in a sample of adult inpatients at a tertiary teaching hospital in the ACT region to provide a baseline for effective monitoring and outcome measures, to determine characteristics associated with malnutrition in this group of patients, and to compare data to historical and subsequent cohorts for prevalence of malnutrition and coding of malnutrition. Based on these data we were then able to estimate the unclaimed annual financial loss of hospital income due to undiagnosed and/or undocumented malnutrition.

## 2. Materials and Methods

### 2.1. Study Population

This cross-sectional, observational study involved the collection of nutritional status data of adult inpatients in the aged care, rehabilitation, surgical, medical, critical care, acute care, orthopaedic, gastroenterology, vascular, neurology, renal, respiratory, cardiology, mental health, and oncology wards at a tertiary teaching hospital.

Participant recruitment was on a voluntary basis. Prior to data collection all participants received a copy of an information sheet and consent form. Patients over the age of 18 years admitted to the hospital over the study days were eligible for inclusion. Exclusion criteria were moderate-severe cognitive problems (as documented in the medical notes), no conversational English, day patients, terminal medical illness, and patients who were antenatal, postnatal, or determined as unfit by medical officers. If a participant was identified as malnourished then this was documented in their medical notes and they were referred to the nutrition department for nutrition intervention. Data collection took place over two consecutive days in June 2012. All eligible patients were invited to participate. The research was approved by the ACT Government Health Directorate Human Research Ethics Committee (ETH6.11.126 and ETHLR.13.222), and informed written consent was obtained from all participants.

### 2.2. Nutrition Assessment

**Figure 1:**
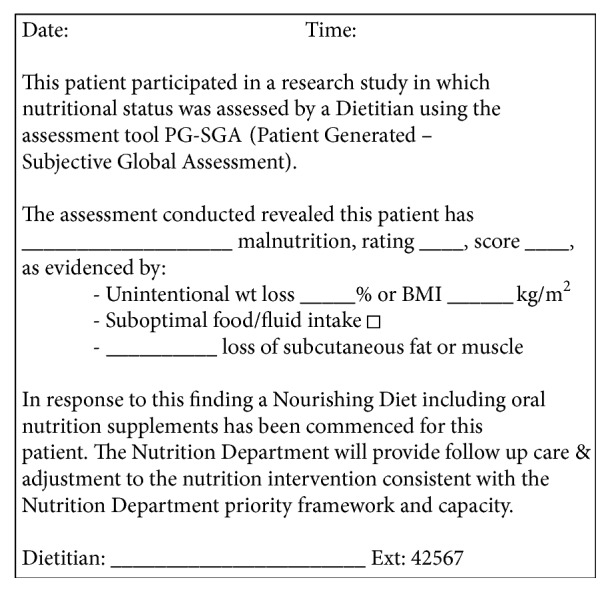
Preprinted malnutrition sticker used for point prevalence study.

Nutritional status of subjects was assessed using the scored Patient-Generated Subjective Global Assessment (PG-SGA) tool [18] which is a validated tool used in the acute care setting [1]. It enables dietitians to determine nutritional status based upon a medical assessment and physical examination. Each participant was classified using a global category rating as either well-nourished (SGA A), moderately or suspected of being malnourished (SGA B), or severely malnourished (SGA C), and a total PG-SGA score was calculated. This score provides a guideline to the level of nutrition intervention required, with the higher the score, the greater the risk of malnutrition [18]. A score ≥ 9 indicates a critical need for improved symptom management and/or nutrient intervention options [18]. This was documented accordingly in the medical records by dietitians completing the nutrition assessment. Standardised preprinted stickers were used for this purpose to help identify patients to the medical coders and to standardise dietetic practice (Figure 1). The sixteen dietitians who were involved in collecting the data in this setting currently use the PG-SGA tool in the routine care of their patients. Standardised training in performing the PG-SGA was conducted for dietitians involved in the data collection by one of the authors (JK). Interrater reliability of all 16 dietitians involved in the data collection was determined and showed good agreement with the use of the PG-SGA (Intraclass correlation (ICC) = 0.901; *p* < 0.001).

### 2.3. Medical Records Audit

Following the data collection, a retrospective review of the medical records of study participants was conducted by the authors to provide information regarding length of stay, DRG allocation, documentation of malnutrition, and dietetic intervention. This was to determine if the study participants who were diagnosed as malnourished in this study were documented as malnourished by the dietitians and coded as malnourished by the medical coders. These data were then compared to the medical records of two historical control groups (June 2011 and March 2012) and two subsequent cohorts (September 2012 and June 2013) to determine the proportion of patients coded as being malnourished prior to our study and after our study. All patient separations within these months using the same inclusion criteria were included in the data collection. A patient separation can be defined as, “the process by which an episode for an admitted patient ceases [19].”

Unclaimed potential reimbursements to this hospital (per annum) were calculated based on previous studies [6, 14], where Agarwal et al. [7] determined the average reimbursement per patient whose DRG changed because malnutrition coding was AU$3470. Agarwal et al. [7] calculated that malnutrition coding for approximately 20% of malnourished patients led to an increase in financial reimbursement of AU$3470 per patient. This figure of 20% is based on the average percentage of patients for whom the DRG changed because of malnutrition coding in studies by Lazarus and Hamlyn [6] and Gout et al. [14] and the amount of $3470 based on the average hypothetical reimbursement per patient from the above-mentioned studies [6, 14]. The malnutrition prevalence data collected in June 2012 was used for this calculation which is comparable to these previous studies for the purpose of calculating unclaimed potential financial reimbursements.

### 2.4. Statistical Analysis

Data were entered into IBM SPSS Version 21.0 software (IBM Corp., released 2012, IBM SPSS Statistics for Windows, version 21.0, Armonk, NY: IBM Corp). Patient characteristics were reported as frequencies, means, and standard deviations. Nonparametric techniques were principally used to describe patient characteristics. Associations between gender and continuous variables were assessed using the independent* t*-test. Associations between continuous variables and malnutrition prevalence were assessed using the One-Way ANOVA test. The Mann–Whitney* U* test was used to examine differences in variables between the groups of patients according to malnutrition status as assessed by the PG-SGA. For this purpose groups SGA B and SGA C (i.e. malnourished patients) were combined for statistical purposes. A *p* value of less than 0.05 was considered to be statistically significant. Intraclass correlation was used to show the measure of agreement between multiple raters.

## 3. Results

One hundred and eighty-nine patients participated in the study over the two days. There were 434 adult inpatients at the hospital over the study period, and 163 were excluded (100 because of being determined unfit by medical officers, 22 because of contact precautions, 18 because of being antenatal/postnatal, 8 because of having no conversational English, 7 because of having moderate-severe cognitive problems, 6 because of having terminal medical illness, and 2 because of being aphasic). Of the 271 eligible patients approached to participate, 75 declined and 7 were away from their room at the time of data collection. Overall the response rate was 73.1%.

One hundred and seventeen participants (62%) were male, and 72 (38%) were female. The age range of the 189 participants was 18–97 years (mean age 62.9 ± 17.9). Fifty-two percent of the participants were over the age of 65 years. The baseline characteristics of our participants are summarised in Table 1.

Forty seven percent (*n* = 88) of patients were classified as well-nourished, 47% of patients (*n* = 89) were classified as moderately or suspected of being malnourished, and 6% (*n* = 12) of patients were classified as severely malnourished. By ward, 96% of the participants from the oncology ward, 83% of the participants from the aged care ward, and 79% of the participants from the respiratory ward were found to be moderately or severely malnourished.

The characteristics of patients according to malnutrition status were assessed according to the Patient-Generated Subjective Global Assessment (PG-SGA) tool as shown in Table 2. There was no association between malnutrition status and gender (*p* = 0.051). There was a significant association between malnutrition and increasing age (*p* = 0.040). There was a significant difference between mean PG-SGA scores for each of the SGA classifications (*p* < 0.001), with the severely malnourished having the highest scores. There was also an association between SGA groups and body mass index (BMI), with the higher the SGA classification, the lower the BMI (*p* < 0.001). There was a significant difference in length of stay between well-nourished patients (SGA A) (median = 8, *n* = 88) and malnourished patients (SGA B and SGA C) (median = 14, *n* = 101),* U* = 2961,* Z* = −3.96, *p* < 0.001, and *r* = −0.28. On the study days in June 2012, malnutrition was coded in 98.2% (*n* = 99) of patients assessed as malnourished. Eighty-five percent of malnourished patients were coded as having been seen by a dietitian.

The results of the medical history audit of patients that was conducted on patient separations in June 2011, March 2012, September 2012, and June 2013 are shown in Table 3. Between 0.9 and 5.4% of patients were coded as malnourished. The number of patients coded as malnourished was higher for the months of September 2012 and June 2013 (after the point prevalence study in June 2012), being 5.4% and 4.4%, respectively. The number of malnourished patients seen by a dietitian increased from 74% to 89% between June 2011 and June 2013. The mean age of patients coded as being malnourished over all months was greater than 65 years. In June 2011 the mean age of patients coded as being malnourished was 69.1 ± 18.2; in March 2012 it was 70.1 ± 14.3, in September 2012 it was 71.2 ± 17.1, and in June 2013 it was 66.4 ± 19.9.

Extrapolating from Agarwal et al. [7], we can estimate the unclaimed potential reimbursements per annum to this hospital in the ACT region of Australia to be AU$8,536,200. This figure was calculated considering that the average number of patient separations per month is 1938. If 53% of these patients are malnourished each month, then an estimated 1027 patients would be malnourished each month. Previous studies [6, 14] have found that 20% of the malnourished patients led to an average increase in financial reimbursement of $3470; therefore 20% of 1027 = 205 and then 205 × $3470 = AU$711,350 per month and therefore AU$8,536,200 per year.

## 4. Discussion

In this observational study, we found the prevalence of malnutrition to be 53% amongst the adult inpatients, which is the highest prevalence of malnutrition recorded in an Australian study, equivalent to research conducted by Thomas et al. in 2007 [8]. Previous other Australian studies have found malnutrition to be prevalent in 12–42% of patients [3–7]. Within the hospital population, older adults are particularly vulnerable. Our study showed that there was a significant association between malnutrition and increasing age, decreasing body mass index (BMI), and an increased length of stay. Fifty-two percent of participants were over the age of 65, with the average age of participants (approaching this age group) being sixty-three years of age. The association between malnutrition status and gender was not significant, however there was a trend and our results did show that more females were malnourished compared with males. Previous research by Banks et al. [3] and Middleton et al. [5] found that gender did not have an effect on nutritional status in acute facilities.

When we compared our study data to two historical control groups and two subsequent control groups we found that between 0.9% and 3.8% of patients were coded as being malnourished before the study and 4.4%–5.4% of patients were coded as being malnourished in the subsequent control groups. These figures are significantly lower than the 52% of patients that were coded as malnourished from the point prevalence study data (98% of the patients diagnosed as malnourished (53%) were coded as being malnourished in the medical records). It is difficult to ascertain whether this difference in malnutrition coding figures was due to lack of identification, documentation, or coding in the control groups, but considering the total number of occasions of service for dietitians documented in the medical notes (Table 3) and the high accuracy of the medical coders found in this study (98%), it is most likely to be primarily due to lack of identification (i.e., diagnosis) of malnutrition. In our point prevalence study, we used standardised preprinted stickers to help identify malnourished patients to coders and to standardise dietetic practice, which may have increased the coding of the malnourished patients by the medical coders. These data also showed that, since the point prevalence study, there has been an increase in the number of patients coded as malnourished and an increase in the number of malnourished patients seen by a dietitian, which shows that point prevalence study increased the awareness of malnutrition by the hospital dietitians and increased the diagnosis and documentation of malnutrition. The increase in the number of patients coded as malnourished in between June 2011 (0.9%) and March 2012 (3.8%) (prior to the study) may be a result of the clinicians' altered behaviour resulting from the awareness of the upcoming study which occurred in June 2012 (known as the Hawthorne Effect) [20].

It is also important to recognise that the point prevalence study was conducted under study conditions, where 16 dietitians were conducting assessment, all eligible patients were assessed, and malnutrition stickers were used. These are ideal conditions and may vary to “usual care,” where factors affecting the identification of malnutrition may include poor completion of malnutrition screening by nursing staff, poor referral rates to the dietitian, and patients who are discharged before being seen by the dietitian for assessment. The historical and subsequent cohorts were representative of “usual care” which may also account for some of the differences in coding of malnutrition between groups.

We estimated the unclaimed potential reimbursements per annum to this hospital to be AU$8,536,200. This additional funding to the health facility could make a valuable contribution to the increased costs associated with treating these malnourished patients. For example, if these costs were recovered, a portion of this additional funding could be directed to funding the employment of additional Accredited Practising Dietitians (APDs) to assist with the identification and management of patients with malnutrition, which may improve clinical outcomes.

Malnutrition as a health issue in the hospital setting was first documented in the medical literature over forty years ago [21]. Since then, many studies have been published on the topic of hospital malnutrition [3, 14]. Despite the awareness of this issue in the medical literature, this study shows that malnutrition continues to go unrecognised or undiagnosed in the acute care setting. This finding is supported by previous Australian studies [5, 6, 22–24]. Our findings highlight the importance of regular dietetic assessment amongst high-risk patients to ensure that malnutrition is recognised and diagnosed. This may then facilitate timely and adequate patient care. It is also imperative that correct documentation of malnutrition gets coded in the medical notes so that an appropriate DRG can be determined which influences reimbursement of funds to the health facility.

The reported prevalence rate of malnutrition in this study may be an underestimation of this clinical issue, as a result of the exclusion criteria used. Patients with dementia were excluded who are a high-risk group for malnutrition [25]. Including patients with dementia would require informed consent from family members and would require a family member or caregiver to be interviewed to get reliable data on elements of the PG-SGA. These patients may have a higher prevalence of malnutrition and their inclusion would provide a more accurate picture of malnutrition in the acute care setting in the ACT region of Australia.

A contributing factor towards the high prevalence of malnutrition found in this study may be a result of the increase in acuity of patient admissions over time. For example, in 2000-2001, the average length of stay in an Australian Public Hospital was 3.7 days [26]. The most recent data from 2014-2015 shows that this has now increased to 5.7 days [27]. In addition, with our ageing population in Australia, there has been an increase in hospitalisation for people aged between 65 and 74 years by an average of 6.0% each year between 2010-2011 and 2014-2015, which is faster than the population growth for this age group which was 4.6% each year for the same period [27]. During 2014-2015, people aged 65 years and over accounted for 41% of hospitalisation in Australia and 49% of patient days, when they currently make up 15% of Australia population [27]. With the proportion of our ageing population continuing to increase, this may impact on the prevalence of malnutrition in the hospital setting.

Using validated nutrition assessment tools to diagnose malnutrition in the acute care setting is of paramount importance. In the acute care setting, valid nutrition assessment tools include the Subjective Global Assessment (SGA) [28], the PG-SGA [18], and the Mini-Nutritional Assessment (MNA) (in older adults only) [29]. Diagnosing malnutrition in a timely fashion enables a dietetic referral to be triggered so that the patient can receive appropriate dietetic input. It has been suggested that validated nutrition assessment tools be used at baseline and then on a monthly basis to provide pre- and postintervention comparisons [1].

Mitchell and Porter [30] highlight the lack of evidence in the care of malnourished hospital adults, which limits the ability of clinicians and healthcare managers to make informed, cost-effective treatment decisions for this vulnerable group of patients. There is also an evidence gap regarding the economic considerations of nutrition assessment. We do not currently know if nutrition assessment is cost-effective. A full cost-effectiveness study on the benefits of nutrition assessment would make a valuable contribution to malnutrition research.

In conclusion, this study has highlighted that malnutrition continues to be underdiagnosed in the acute care setting in Australia, with older adults being particularly vulnerable. Diagnosing malnutrition using validated assessment tools and documenting and coding accordingly may increase potential reimbursements for hospitals which enables funding to be directed towards better care. Using standardised preprinted stickers may assist in identifying malnourished patients to coders and standardising documentation by dietitians. Malnutrition has been strongly associated with adverse clinical outcomes such as an increased length of stay and higher rates of medical complications. Not diagnosing malnutrition presents a high risk to patients and is a lost opportunity for financial reimbursement for the increased costs associated with the care of these vulnerable patients.

## Figures and Tables

**Table 1:** Demographic variables of 2012 point prevalence study population (*n* = 189).

Patient characteristic	Number
Age (years)	62.9 ± 17.9 (age range: 18–97)
Gender	
Male	117 (62%)
Female	72 (38%)
Nutritional status	
SGA A (well-nourished)	88 (47%)
SGA B (suspected or moderately malnourished)	89 (47%)
SGA C (severely malnourished)	12 (6%)
PG-SGA score (median)	7 (PG-SGA score range: 0–25)

**Table 2:** Characteristics of patients according to malnutrition status as assessed by the Patient Generated Subjective Global Assessment (PG-SGA) tool (2012 study data, *n* = 189).

	SGA A (well-nourished)	SGA B (suspected or moderately malnourished)	SGA C (severely malnourished)	*p* value
Gender (*n*, (%))				0.051
Male	62 (33%)	47 (25%)	8 (4%)	
Female	26 (14%)	42 (22%)	4 (2%)	
Age (years)	59.4 ± 18.7	66.0 ± 17.0	66.0 ± 13.9	0.040
PG-SGA score	3.5 ± 2.6	12.0 ± 4.1	18.6 ± 3.2	<0.001
Body mass index (kg/m^2^)	29.3 ± 7.8	25.3 ± 6.7	22.7 ± 5.9	<0.001

**Table 3:** Medical history audit of patients assessed as malnourished.

Date	Number of patient separations	Number of patients coded as malnourished (%)	Mean age of patients coded as malnourished (range)	Number of malnourished patients seen by a dietitian (%)	Number of patients seen by a dietitian for any condition (excluding malnutrition)
June 2011	1963	19 (0.9)	69.1 ± 18.2 (30–93)	14 (74)	210/1963 (10.6%)
March 2012	2006	76 (3.8)	70.1 ± 14.3 (25–94)	64 (84)	154/2006 (7.7%)
Sept 2012	1906	103 (5.4)	71.2 ± 17.1 (18–99)	89 (86)	94/1906 (4.9%)
June 2013	1876	82 (4.4)	66.4 ± 19.9 (18–96)	73 (89)	95/1876 (5.1%)
